# HLA Haplotypes and Genotypes Frequencies in Brazilian Chronic Periodontitis Patients

**DOI:** 10.1155/2015/481656

**Published:** 2015-08-03

**Authors:** Emília Ângela Sippert, Cléverson de Oliveira e Silva, Christiane Maria Ayo, Silvia Barbosa Dutra Marques, Jeane Eliete Laguila Visentainer, Ana Maria Sell

**Affiliations:** ^1^Post Graduation Program of Biosciences Applied to Pharmacy, Biomedicine and Clinical Analysis Department, Maringa State University, 87020900 Maringá, PR, Brazil; ^2^Dentistry Department, Maringa State University, 87020900 Maringá, PR, Brazil; ^3^Hemocentro of Campinas, HLA Laboratory, University of Campinas, 13083878 Campinas, SP, Brazil; ^4^Basic Health Sciences Department, Maringa State University, 87020900 Maringá, PR, Brazil

## Abstract

Human leukocyte antigens (HLA) have a pivotal role in immune response and may be involved in antigen recognition of periodontal pathogens. However, the associations of HLA with chronic periodontitis (CP) have not been previously studied in the Brazilian population. In an attempt to clarify the issue of genetic predisposition to CP, we examined the distribution of HLA alleles, genotypes, and haplotypes in patients from Southern Brazil. One hundred and eight CP patients and 151 healthy and unrelated controls with age-, gender-, and ethnicity-matched were HLA investigated by polymerase chain reaction with sequence specific oligonucleotides. To exclude smoking as a predisposing factor, statistical analyses were performed in the total sample and in nonsmoking individuals. The significant results showed a positive association of the A^∗^02/HLA-B^∗^40 haplotype with CP (total samples: 4.2% versus 0%, *P*_*c*_ = 0.03; nonsmokers: 4.3% versus 0%, *P*_*c*_ = 0.23) and a lower frequency of HLA-B^∗^15/HLA-DRB1^∗^11 haplotype in CP compared to controls (total samples: 0.0% versus 4.3%, *P*_*c*_ = 0.04; nonsmokers: 0 versus 5.1%, *P*_*c*_ = 1.0). In conclusion, the HLA-A^∗^02/B^∗^40 haplotype may contribute to the development of CP, while HLA-B^∗^15/DRB1^∗^11 haplotype might indicate resistance to disease among Brazilians.

## 1. Introduction

Chronic periodontitis (CP) is a common complex disease of the oral cavity that is characterized by an inflammatory response to commensal and pathogenic oral bacteria [1, 2]. Due to bacterial infection, periodontal tissues become inflamed and are slowly destroyed by the action of the inflammatory process. If the disease is left untreated, teeth lose their ligamentous supporting structure to the alveolar bone, the alveolar bone is resorbed, and the teeth become mobile, finally resulting in teeth loss [1]. CP is considered the main cause of tooth loss among adults and is associated with severe quality of life impact [3].

The inflammatory response of the periodontal tissues to infection is influenced by environmental factors as well as by genetic factors [4, 5]. It is estimated that 50% of the expression of periodontitis in CP could be attributed to genetic factors [5]. The observation that periodontitis is a complex disease entity with a multifactorial etiology has led to the search for risk factors that predispose to periodontitis in general as well as distinctive risk factors that might predispose to different clinical presentations of this group of diseases.

The human leukocyte antigens (HLA) play an important role in immune responsiveness and may be involved in antigen recognition of periodontal pathogens [6]. These cell-surface molecules have a key role in antigen presentation and activation of T cells. The polymorphisms of HLA can directly affect the binding capability of antigen peptides and thus affect the antigen-specific T-cell response [7]. Hence, these polymorphisms could represent an important susceptibility or resistance factor to periodontitis.

For many years, researchers have periodically screened populations of patients with different forms of periodontitis for associations with HLA antigens [8–14] and consistent results in relation to CP could not be obtained up to now. This study aimed to investigate differences in allelic group, genotype, and haplotype frequencies of HLA classes I and II in a sample of Brazilian patients with CP compared with a control group without CP.

## 2. Materials and Methods

### 2.1. Sample Selection

Between January and September 2012, a total of 259 individuals were selected from those who sought dental treatment at the dental clinics of Maringa State University (UEM) and Inga University (UNINGÁ) at Maringá, PR, Brazil (north/northwest region of the State of Parana, located in the southern region of Brazil, between 22°29′30′′–26°42′59′′S and 48°02′24′′–54°37′38′′W). Males and females, ethnically similar, aged over 34 years and with at least 20 teeth in the buccal cavity participated in this study. The criteria for exclusion were as follows: individuals with acute infections or diseases with known associations to HLA alleles such as diabetes, rheumatic diseases, systemic lupus erythematosus or narcolepsy, use of antibiotics during the last six months, and chronic usage of anti-inflammatory drugs or lactations and those who were pregnant.

After taking the patient's history, clinical periodontal examinations were conducted by two examiners. Clinical parameters of probing depth (PD) and clinical attachment level (CAL) were examined at six sites (mesiovestibular, vestibular, distovestibular, mesiolingual, lingual, and distolingual) of each tooth, as was bleeding on probing (BOP). After the periodontal examination, participants were categorized into two different groups: the CP group (*n* = 108) composed of individuals who had at least 5 sites in different teeth with PD ≥ 5 mm, CAL ≥ 3 mm, and more than 25% of BOP; and the control group (*n* = 191), formed by individuals who did not have sites with reduced CAL, displayed a PD of less than 4 mm, and exhibited less than 25% of BOP. Therefore, the control group was matched to the case group according to epidemiological characteristics such as ethnicity, gender, and geographical region. Information on the habit of smoking was obtained by interviewing the individual (anamnesis).

### 2.2. Ethics Information

All individuals who agreed to participate in this research were informed regarding the nature of the study and signed an informed consent form authorizing the use of their samples in the study, which was approved by the Ethics Committee for Human Research at Maringa State University (UEM-N°.719/2011, 02/12/2011).

### 2.3. Sample Collection and DNA Extraction

Blood samples (4 mL) were collected from the subjects in tubes containing anticoagulant (EDTA) and centrifuged at 210 g for 15 minutes, and the buffy-coat was conserved at −20°C until use. The genomic DNA was extracted using the salting-out method described by Miller et al. [15]. The concentration and quality of the DNA were analyzed by optical density in a Thermo Scientific Nanodrop 2000 apparatus (Wilmington, USA).

### 2.4. HLA Genotyping

HLA typing (HLA-A, HLA-B, HLA-C, DRB1, DQA1, and DQB1) was carried out using the polymerase chain reaction-sequence specific oligonucleotides technique (PCR-SSO; One Lambda, Canoga Park, CA, USA), low resolution to HLA-A, HLA-B, HLA-C, and DRB1, and high definition to HLA-DQA1 and DQB1. First, target DNA was PCR-amplified using group specific primers set, after the amplified product was biotinylated, which allowed later detection using R-phycoerythrin-conjugated streptavidin (SAPE), and hybridized with microspheres linked to specific conjugated fluorescent probes for HLA allele groups (One Lambda, Canoga Park, CA, USA). The fluorescent intensity varied based on reaction outcome and was expected to be 1000 or above for control positive probes. Reaction readings were carried out by flow cytometry using Luminex technology (One Lambda). Samples were analyzed through the HLA FUSION software (One Lambda Inc., San Diego, CA, USA).

### 2.5. Statistics

HLA specificity frequencies between the groups were compared using Fisher exact test. A two-sided *P* value (*P*) of <0.05 was considered statistically significant. Odds ratio (OR) values with a 95% confidence interval (95% CI) were also calculated to evaluate the risk of the individual developing the disease when having a particular HLA type. All the statistical analyses were performed using the *R* statistical environment [16]. However, to account for multiple comparisons, the observed *P* values were corrected (*P*_*c*_) for the number of alleles when one locus was considered alone (Bonferroni correction). As the gametic phase of alleles from the different loci was not known and segregation analysis into families was not carried out, haplotypes and the calculation of their frequency were determined by the likelihood ratio test, through the Arlequin statistical software program [17]. Hardy-Weinberg equilibrium [18] was achieved by calculating the expected genotype frequencies and comparing them to the observed values, also using Arlequin statistical software program [17]. To exclude smoking as a predisposing factor statistical analyses were performed in the total sample (smokers, ex-smokers, and nonsmokers: patientsversus control) as well as in nonsmoking patients versus nonsmoking controls. Only the haplotypes with frequencies above 1.0% were considered in this study.

## 3. Results

The study involved 259 individuals, 108 of whom were patients with CP, while 151 controls were healthy individuals. At the time of sample collection, the ages of the patients and controls ranged between 34 and 81 years (47.22% males and 52.78% females by patients and 35.10% males and 64.90% females by controls). Regarding the ethnic background, 59.26% of the patients with CP were reported to be Caucasians, 27.78% racially mixed, and 12.96% Afro-Brazilian. Concerning the control individuals, 69.54% were reported to be Caucasians, 23.84% racially mixed, and 6.62% Afro-Brazilian. No differences were observed between patients and controls according to gender, age, and ethnicity. Smoking was associated with CP and was more frequent in patients than in controls (*P* = 0.043; OR = 2.12; 95% CI = 1.02–4.51) as well as in ex-smokers compared to controls (*P* = 0.001, OR = 2.7; 95% IC = 1.46, 5.05) (Table 1).

In all groups, the distribution of HLA genotypes was confirmed to be in Hardy-Weinberg equilibrium (*P* ≥ 0.05 in the patients and in the controls).

The most common HLA types were the following: HLA-A^*∗*^01, ^*∗*^02, ^*∗*^03, and ^*∗*^24 and HLA-B^*∗*^15, ^*∗*^35, ^*∗*^44, and ^*∗*^51, similar to other previous reports in the north/northwest region of the State of Parana [19]; this was an important control to demonstrate that the population was representative.

Table 2 presents the HLA classes I and II group allele frequencies distribution only for the significant differences between patients and controls. When comparing the HLA class I frequencies between CP and controls, HLA-A^*∗*^32 had a lower frequency in patients with CP (smokers, nonsmokers, and ex-smokers) and HLA-A^*∗*^02 in nonsmokers group; HLA-B^*∗*^40 had a higher frequency in CP total patients and also in nonsmokers patients group; no significant differences were found for HLA-C allelic groups. In relation to class II, HLA-DRB1^*∗*^08 allelic group and DQB1^*∗*^06:09 were overrepresented in patients compared to controls, and HLA-DQB1^*∗*^06:01 was less frequent in patients compared with controls; however, neither of these associations was confirmed in the nonsmoker group.

Differences between CP and controls were found comparing the HLA genotypes frequencies for homo- and heterozygosis. HLA-C^*∗*^03-C^*∗*^06 genotype was increased in CP, whereas HLA-DQB1^*∗*^03:01 homozygous genotype and DQB1^*∗*^03:01/DQB1^*∗*^03:02 were significantly less frequent in CP (Table 3). After Bonferroni correction, the associations lost the significance.

Regarding haplotype frequencies (Tables 4 and 5) patients with CP had significantly higher frequencies for HLA-A^*∗*^02/HLA-B^*∗*^40 and a lower frequency for HLA-B^*∗*^15/HLA-DRB1^*∗*^11. Furthermore, patients with CP (only total samples) had higher haplotypes frequencies for B^*∗*^18/C^*∗*^12, B^*∗*^51/C^*∗*^01, B^*∗*^50/DRB1^*∗*^04, DQA1^*∗*^01:02/DQB1^*∗*^06:09, and DRB1^*∗*^13/DQA1^*∗*^01:02/DQB1^*∗*^06:09 (Table 4); for nonsmokers B^*∗*^44/DRB1^*∗*^07, B^*∗*^35/DRB1^*∗*^11, and B^*∗*^14/DRB1^*∗*^01 were more frequent and HLA-A^*∗*^02/HLA-B^*∗*^35 was less frequent in patients compared to controls (Table 5); A^*∗*^03/B^*∗*^51, A^*∗*^26/B^*∗*^38, B^*∗*^40/C^*∗*^03, and B^*∗*^40/DRB1^*∗*^07 (Tables 4 and 5) had higher frequencies for both groups, although for all of them the significance was lost after Bonferroni correction.

## 4. Discussion

While many studies have shown associations of HLA polymorphisms with aggressive periodontitis in different populations [8, 10–14, 20–24] there are few studies of HLA association with CP and the results are inconsistent [9–11, 20, 25–27]. To the best of our knowledge, this is the first study of HLA association with CP in the Brazilian population. Our results provide evidence that HLA classes I and II are associated with chronic periodontitis.

In general, discrepancies in results are observed in HLA case-control studies, and these discrepancies could be caused by differences in the choice of controls, disease diagnostic, racial background, and statistical analyses [28]. In this study, in order to avoid bias in the final results, the control population was selected after clinical examination with the same criteria used for patient exclusion, such as gestation and manifestations of infectious disease or known medical conditions that may contribute to development of CP. Patients with aggressive periodontitis were also excluded. Other confounding variables as gender, age, and ethnicity were considered. Gender and age ratio was similar between patients and controls. Reichert et al. [20] warned against the fact that gender could represent a confounding variable that should be considered in HLA and periodontitis studies; however, as HLA antigen expression has been reported to not vary as a function of gender [29, 30], this expression was probably not taken into consideration in many of the earlier studies on HLA association for periodontal diseases [10, 11, 13, 27, 31]. Age was not related to HLA expression but can be related to the disease, although severity and prevalence were mostly related to past disease history, social and behavior factors [32], and altered inflammatory responses [33].

Important to emphasize is that HLA varies according to population and ethnic group and that Brazil has an admixed population. The population in Parana has been well defined regarding ethnicity and HLA distribution: in accordance with HLA phenotypic classification, the white population (majority) is predominantly of European origin (80.6%), with a smaller contribution of African (12.5%) and Amerindian (7.0%) genes [34]. Thus, regarding ethnic backgrounds, periodontitis patients and controls were similar, not representing a confounding variable. For this reason added to the high polymorphism of HLA, stratification by ethnicity was not realized.

In this study, as expected, smoking was associated with CP and having stopped smoking maintained susceptibility to disease. As smoking is a risk factor for the onset and progression of CP and the habit can obscure genetic risk factors [35] we analyzed nonsmoking patients and nonsmoking controls in addition to the total group (smokers, ex-smokers, and nonsmokers).

In the current study some trends to associations of HLA class I antigens with CP were found. HLA-A^*∗*^32 was less frequent in total samples and HLA-A^*∗*^02 was less frequent in nonsmoker patients representing possible protection to CP; however, significance was lost after Bonferroni correction. Added to these results, the HLA-A^*∗*^02/B^*∗*^35 haplotype was significantly less frequent in nonsmoker patients. Similar to our results, pioneering and recent investigations involving periodontitis and HLA have also found HLA^*∗*^02 associated with protection to periodontitis [25, 27, 36, 37]. Thus, our results indicate a protective role of HLA-A^*∗*^02. A2 could yield an efficient antimicrobial T-cell response reducing the disease [25].

Otherwise, HLA-B^*∗*^40 was more frequent in both groups representing a risk effect against CP, although significance was also lost after Bonferroni correction. However, HLA-A^*∗*^02/B^*∗*^40 haplotype was significantly associated with susceptibility to CP. Added to this result, HLA-B^*∗*^40/C^*∗*^03 and HLA-B^*∗*^40/DRB1^*∗*^07 haplotypes tend to be associated with CP susceptibility. As HLA-A^*∗*^02/B^*∗*^40 was in linkage disequilibrium (Δ′ > 0.70, for all analyzed population: patients and controls) and HLA-A^*∗*^02 has been associated as a protection factor, the susceptibility found for patients with CP may be related to HLA-B^*∗*^40. Individuals carrying B^*∗*^40 (B60 and B61 antigens) had a three-fold higher risk of developing the disease. Contrarily, in another reporter, HLA-B^*∗*^40 was associated with a lower clinical attachment loss in CP; however, this association was lost after Bonferroni correction [31].

Taking into account other haplotypes that were significantly associated in our population, the HLA-B^*∗*^15-DRB1^*∗*^11 combination must be highlighted conferring protection against CP in the total group of patients. HLA-B^*∗*^15-DRB1^*∗*^11 was in linkage disequilibrium (Δ′ > 0.80 for all analyzed population). HLA-B^*∗*^15 could be associated with the disease: no associations between DRB1^*∗*^11 and CP have been previously reported; however, in agreement with our results, Mauramo et al. [31] found that patients expressing HLA-B^*∗*^15 had less clinical periodontal disease manifestation and better periodontal health over a long period of time. HLA-B^*∗*^15 positive individuals might have a somewhat peculiar genetic response towards periodontal bacteria challenge contributing to CP development [31].

Another haplotype that lost the significance after Bonferroni correction in our study but must be highlighted was HLA-B^*∗*^50/DRB1^*∗*^04. This haplotype was a susceptibility factor to CP in our population and it is possible that the effect was associated with DRB1^*∗*^04. According to previous reports, and in agreement with our results, HLA-DRB1^*∗*^04 was associated with risk to CP [38, 39] and aggressive periodontitis [9, 12, 23, 30], as well as their alleles [8, 30]. DRB1^*∗*^04:04 was considered a risk factor for bone loss [30, 40]. Contrarily, in a recent investigation, HLA-DRB1^*∗*^04 or HLA-DRB1^*∗*^04/DRB4^*∗*^(DR53)/DQB1^*∗*^03:02 haplotype had a decreased colonization risk of* Aggregatibacter actinomycetemcomitans* [9]. DRB1^*∗*^04 was an immunogenetic susceptibility factor for type 1 diabetes [41] and for rheumatoid arthritis [30]; however, both diseases were included in our exclusion criteria.

According to other association studies between periodontitis and HLA, a meta-analysis focusing on Caucasian case-control studies conducted by Stein et al. [25] demonstrated no associations between HLA and CP, although for aggressive periodontitis HLA-A^*∗*^09 and B^*∗*^15 appeared to represent susceptibility factors and HLA-A^*∗*^02 and B^*∗*^05 were potential protective factors. Other HLA-A, HLA-B, and HLA-C associations with periodontal disease previously described in the literature were as follows: susceptibility mediated by HLA-A^*∗*^11, A^*∗*^29, B^*∗*^14, and C^*∗*^08 and protection by HLA-A^*∗*^03, HLA-A^*∗*^31 and -A^*∗*^30/A^*∗*^31 genotype observed in German patients with CP [11] and HLA-B^*∗*^57 as a protecting factor in German patients with CP or generalized aggressive periodontitis [9]; HLA-A^*∗*^02 and HLA-B^*∗*^05 associated with protection in the CP patients from USA [27, 36, 37]; HLA-A^*∗*^09 positively associated with CP from France [26]; and HLA-B^*∗*^51 related to fewer deep periodontal pockets in the CP in Swiss adults. HLA-DQA1^*∗*^03 and HLA-DRB1^*∗*^04 were higher and HLA-DQB1^*∗*^06:03 might have protective effects against aggressive periodontitis in Iranian patients [8].

A possible role of HLA in the immune response and development of periodontal disease may be related to their ability to bind some processed peptides from bacteria antigens and expressing them on the surface of antigen presenting cells (peptide-HLA class II) or target cells (peptide-HLA class I) in order to present them to T cells (CD4 or CD8) [42]. The binding capacity of the bacteria peptide depends on HLA allotypic structure of their paratope. Failures in this link capacity can compromise the immune response and could be a risk of disease. The large individual capacity of immune response occurs due to the fact that HLA is highly polymorphic: each gene has multiple alleles and each individual has many expressed genes. These preliminary results indicate that HLA haplotypes might be involved in the susceptibility or risk for periodontal disease. However, further investigations of HLA haplotypes markers in relation to antigenic peptide-binding motifs are necessary in order to understand its relation in periodontitis.

The weaknesses of the case-control studies were related to the reproduction of results in function of ethnicity background of the individuals, sample size, diagnostic tolls of disease, and HLA genotyping methods. Consequently, no strong association could be found, especially in CP. In addition, CP is a complex and multifactorial disease and several other genetic polymorphisms have been reported to be associated with bacterium response, inflammation, chronicity, and wide ranging systemic effects and disease development, such as* IL1, IL4, TNF, IL8*, and* IL10* cytokines genes, immunoglobulin G Fc receptor (FcyR), and TLR [35, 43–60]. Despite this fact, all information regarding genetic susceptibility to diseases is valuable and can be applied to therapy intervention or individual approach. For better results, we have tried to be very critical in the choice of the study population. CP inclusion criteria and CP and control exclusion criteria were defined in order to avoid confounding factors and were a representative of generally healthy and CP adults. As independent multiple comparisons were carried out and we considered that all genotypes examined had the same chance of being increased or decreased in CP, Bonferroni correction was applied for all significant *P* values.

The present study has potential limitations. We focused on the HLA allele group, not on alleles and epitopes analysis, and split antigens were not investigated. The major limitation of this study is the relatively small sample size regarding the analysis of the nonsmoker group.

In these Brazilian CP patients, the HLA-A^*∗*^02/B^*∗*^40 haplotype was considered a risk factor for CP and HLA-B^*∗*^15/DRB1^*∗*^11 haplotype was considered a protective factor for disease. Susceptibility may possibly be associated with B^*∗*^40 molecules and protection may be associated with B^*∗*^15 and A^*∗*^02. Further investigation relating antigenic peptide-binding motifs and their immunopathogenesis involving CP development should be investigated.

## 5. Conclusion

These results provide evidence that class I and II HLA polymorphisms are associated with chronic periodontitis. HLA-A^*∗*^02/B^*∗*^40 haplotype seems to represent susceptibility factors and HLA-B^*∗*^15/DRB1^*∗*^11 haplotype was potential protective factors against disease.

## Figures and Tables

**Table 1:** Characteristics of patients with chronic periodontitis and controls.

Category	Subcategory	Patients	Controls	Total
		(*N* = 108)	(*N* = 151)	(*N* = 259)
		*n* (%)	*n* (%)	*n* (%)
Gender	Female	57 (52.78)	98 (64.90)	157 (60.62)
	Male	51 (47.22)	53 (35.10)	102 (39.38)

Age	34–49 years	65 (60.18)	97 (64.24)	162 (62.55)
	50–65 years	35 (32.41)	50 (33.11)	85 (32.82)
	66–81 years	8 (7.41)	4 (2.65)	12 (4.63)

Ethnic background	Caucasian	64 (59.26)	105 (69.54)	169 (65.25)
	Afro-Brazilian	14 (12.96)	10 (6.62)	24 (9.27)
	Racially mixed	30 (27.78)	36 (23.84)	66 (25.48)

Smoking	Smoker (**A**)	23 (21.30)	17 (11.26)	40 (15.44)
	Nonsmoker (**B**)	46 (42.59)	108 (71.52)	154 (59.46)
	Ex-smoker (**C**)	39 (36.11)	26 (17.22)	65 (25.10)

**B** versus **A**: *P* ≤ 0.002; OR = 0.32; 95% CI = 0.14–0.68.

**B** versus **C**: *P* ≤ 0.001; OR = 0.29; 95% CI = 0.15–0.54.

**A** versus **C**: *P* = 0.84.

**Table 2:** HLA associations between chronic periodontitis patients and controls.

HLA	Total^**∗**^		*P *	*P*_*c*_	OR (95% CI)	Nonsmokers^**∗∗**^		*P *	*P*_*c*_	OR (95% CI)
	Patients *N* = 216 *n* (%)	Controls *N* = 302 *n* (%)				Patients *N* = 92 *n* (%)	Controls *N* = 216 *n* (%)			
A^*∗*^02	49 (22.7)	86 (28.5)				17 (18.5)	66 (30.6)	0.035	0.63	0.52 (0.26–0.97)
A^*∗*^32	3 (1.4)	15 (5.0)	0.030	0.45	0.27 (0.05–0.97)	1 (1.1)	15 (6.0)			
B^*∗*^40	16 (7.4)	8 (2.6)	0.018	0.38	2.93 (1.16–8.07)	9 (9.8)	7 (3.2)	0.025	0.60	3.22 (1.03–10.55)
DRB1^*∗*^08	17 (7.9)	11 (3.6)	0.048	0.58	2.26 (0.97–5.45)	8 (8.7)	8 (3.7)			
DQB1^*∗*^06:01	0 (0)	7 (2.3)	0.045	0.63	0 (0–0.96)	0 (0.0)	5 (2.3)			
DQB1^*∗*^06:09	5 (2.3)	0 (0.0)	0.012	0.17	undf (1.29–undf)	0 (0.0)	0 (0.0)			

undf = undefined.

^*∗*^Total group (smokers, nonsmokers, and ex-smokers patients and controls).

^*∗∗*^Nonsmoking group (patients and controls).

**Table 3:** HLA genotype frequency associations between chronic periodontitis patients and controls^*∗*^.

HLA genotypes	Patients *N* = 108 *n* (%)	Controls *N* = 151 *n* (%)	*P *	*P*_*c*_	OR (CI 95%)
C^*∗*^03-C^*∗*^06	4 (4.6)	0 (0)	0.012	0.56	undf (1.31–undf)
DQB1^*∗*^03:01-DQB1^*∗*^03:01	0 (0.0)	7 (4.6)	0.044	1.00	0 (0–0.95)
DQB1^*∗*^03:01-DQB1^*∗*^03:02	0 (0.0)	7 (4.6)	0.044	1.00	0 (0–0.95)

undf = undefined.

^*∗*^Total group (smokers, nonsmokers, and ex-smoking patients and controls).

**Table 4:** HLA haplotype associations between chronic periodontitis patients and controls^*∗*^.

HLA haplotypes	Patients *N* = 216 *n* (%)	Controls *N* = 302 *n* (%)	*P *	*P*_*c*_	OR (95% CI)
**A** ^*∗*^ **02/B** ^*∗*^ **40**	**9 (4.2)**	**0 (0.0)**	**<0.001**	**0.03**	undf (2.83–undf)
A^*∗*^03/B^*∗*^51	5 (2.5)	0 (0.0)	0.012	0.36	undf (1.29–undf)
A^*∗*^26/B^*∗*^38	5 (2.3)	0 (0.0)	0.012	0.36	undf (1.29–undf)
B^*∗*^40/C^*∗*^03	12 (5.6)	5 (1.7)	0.022	0.62	3.49 (1.12–12.83)
B^*∗*^18/C^*∗*^12	4 (1.9)	0 (0.0)	0.03	0.93	undf (0.93–undf)
B^*∗*^51/C^*∗*^01	4 (1.9)	0 (0.0)	0.30	0.93	undf (0.93–undf)
**B** ^*∗*^ **15/DRB1** ^*∗*^ **11**	**0 (0.0)**	**13 (4.3)**	**0.001**	**0.04**	undf (0–0.45)
B^*∗*^40/DRB1^*∗*^07	6 (2.8)	0 (0.0)	0.005	0.19	undf (1.67–undf)
B^*∗*^50/DRB1^*∗*^04	4 (1.9)	0 (0.0)	0.030	1.14	undf (0.93–undf)
DQA1^*∗*^01:02/DQB1^*∗*^06:09	5 (2.3)	0 (0.0)	0.012	0.23	undf (1.30–undf)
DRB1^*∗*^13/DQA1^*∗*^01:02/DQB1^*∗*^06:09	5 (2.3)	0 (0.0)	0.012	0.25	undf (1.29–undf)

undf = undefined.

^*∗*^Total group (smokers, nonsmokers, and ex-smoking patients and controls).

**Bold font**: significant after Bonferroni correction.

**Table 5:** HLA haplotype associations between nonsmokers, chronic periodontitis patients, and controls.

HLA haplotypes	Patients *N* = 92 *n* (%)	Controls *N* = 216 *n* (%)	*P *	*P*_*c*_	OR (95% CI)
A^*∗*^02/B^*∗*^40	4 (4.3)	0 (0.0)	0.008	0.23	undf (1.58–undf)
A^*∗*^02/B^*∗*^35	0 (0.0)	11 (5.1)	0.038	1.00	0 (0–0.91)
A^*∗*^03/B^*∗*^51	3 (3.3)	0 (0.0)	0.026	0.75	undf (0.98–undf)
A^*∗*^26/B^*∗*^38	3 (3.3)	0 (0.0)	0.026	0.75	undf (0.98–undf)
B^*∗*^40/C^*∗*^03	7 (7.6)	4 (1.9)	0.019	0.51	4.34 (1.07–20.75)
B^*∗*^44/B^*∗*^07	4 (4.3)	1 (0.5)	0.029	0.78	9.69 (0.94–482.15)
B^*∗*^15/DRB1^*∗*^11	0 (0.0)	11 (5.1)	0.038	1.00	0 (0–0.91)
B^*∗*^40/DRB1^*∗*^07	4 (4.3)	1 (0.5)	0.029	0.81	9.69 (0.94–482.15)
B^*∗*^35/DRB1^*∗*^11	4 (4.3)	1 (0.5)	0.029	0.81	9.69 (0.94–482.15)
B^*∗*^14/DRB1^*∗*^01	4 (4.3)	0 (0.0)	0.008	0.22	undf (1.58–undf)

undf = undefined.
